# A natural experiment to assess how urban interventions in lower socioeconomic areas influence health behaviors: the UrbASanté study

**DOI:** 10.1186/s12889-023-15388-2

**Published:** 2023-03-15

**Authors:** Hélène Charreire, Benoit Conti, Lucile Bauchard, Ndèye Aïta Cissé, Marlène Perignon, Pascaline Rollet, Coline Perrin, Sophie Blanchard, Céline Roda, Thierry Feuillet, Malika Madelin, Vincent Dupuis, Anne-Sophie Evrard, Anne-Peggy Hellequin, Isabelle Coll, Corinne Larrue, Sophie Baudet-Michel, Gabrielle Vernouillet, Fernande Ntsame-Abegue, Isabelle Fabre, Caroline Méjean, Jean-Michel Oppert, UrbASanté Study Group

**Affiliations:** 1 MoISA, Univ Montpellier, CIRAD, CIHEAM-IAMM, INRAE, Institut Agro, IRD, Montpellier, France; 2 LVMT, Univ Gustave Eiffel, Ecole des Ponts, Champs-sur-Marne, France; 3 Innovation, Univ Montpellier, CIRAD, INRAE, Institut Agro, Montpellier, France; 4 Université Paris Est-Créteil, LabUrba, Créteil, France; 5 Université Paris Cité, Health Environmental Risk Assessment (HERA) Team, CRESS, INSERM, INRAE, Paris, France; 6 Université de Caen Normandie, UMR 6266 IDEES CNRS, Caen, France; 7 Université Paris Cité, UMR PRODIG, Paris, France; 8 Sorbonne Université, UMR PHENIX, Paris, France; 9 Université Lyon, Univ Gustave Eiffel, IFSTTAR, Univ Lyon 1, Umrestte, UMR-T9405, Bron, France; 10 UMR LADYSS 7533, Université de Paris Nanterre, Nanterre, France; 11 Université Paris Est Créteil and Université Paris Cité, CNRS, LISA, Créteil, 94010 France; 12 Université Paris Cité, UMR Géographie-cités, Paris, France; 13 Direction de la Santé Publique, Service Parisien Santé Environnement, Ville de Paris, Paris, France; 14 Direction de la Voirie et des Déplacements, Agence de la mobilité, Ville de Paris, Paris, France; 15 Direction de l’Urbanisme, Ville de Paris, Paris, France; 16 Department of Nutrition, Pitié-Salpêtrière Hospital, Assistance Publique-Hôpitaux de Paris (APHP), Sorbonne University, Paris, France; 17 Sorbonne Paris Nord University, INSERM U1153, INRAE U1125, CNAM, Nutritional Epidemiology Research Team (EREN), Epidemiology and Statistics Research Center - University Paris Cité (CRESS), Bobigny, 93017 France

**Keywords:** Natural experiment, Urban intervention, Environmental exposures, Lifestyle behaviors, Self-reported health

## Abstract

**Background:**

Mechanisms underlying the associations between changes in the urban environment and changes in health-related outcomes are complex and their study requires specific approaches. We describe the protocol of the interdisciplinary UrbASanté study, which aims to explore how urban interventions can modify environmental exposures (built, social, and food environments; air quality; noise), health-related behaviors, and self-reported health using a natural experiment approach.

**Methods:**

The study is based on a natural experiment design using a before/after protocol with a control group to assess changes in environmental exposures, health-risk behaviors, and self-reported health outcomes of a resident adult population before and after the implementation of a time series of urban interventions in four contiguous neighborhoods in Paris (France). The changes in environmental exposures, health-related behaviors, and self-reported health outcomes of a resident adult population will be concurrently monitored in both intervention and control areas. We will develop a mixed-method framework combining substantial fieldwork with quantitative and qualitative analytical approaches. This study will make use of (i) data relating to exposures and health-related outcomes among all participants and in subsamples and (ii) interviews with residents regarding their perceptions of their neighborhoods and with key stakeholders regarding the urban change processing, and (iii) existing geodatabases and field observations to characterize the built, social, and food environments. The data collected will be analyzed with a focus on interrelationships between environmental exposures and health-related outcomes using appropriate approaches (e.g., interrupted time series, difference–in-differences method).

**Discussion:**

Relying on a natural experiment approach, the research will provide new insights regarding issues such as close collaboration with urban/local stakeholders, recruitment and follow-up of participants, identification of control and intervention areas, timing of the planned urban interventions, and comparison of subjective and objective measurements. Through the collaborative work of a consortium ensuring complementarity between researchers from different disciplines and stakeholders, the UrbASanté study will provide evidence-based guidance for designing future urban planning and public health policies.

**Trial registration:**

This research was registered at the ClinicalTrial.gov (NCT05743257).

## Background

It has become widely accepted that overall health and quality of life result from a complex interplay between individual (e.g., age, gender, and socioeconomic position) and contextual built and social characteristics of the environment in which individuals live (e.g., transport infrastructures, land use, food environment, and area-level deprivation) [1, 2]. Thus, urban planning choices can affect the health and well-being of the population and contribute to reducing or, in contrast, enhancement of social health inequalities. The impact of urban redevelopment in a neighborhood can vary considerably depending on the socioeconomic characteristics of populations and environments: in some cases, it may increase social health-related inequalities [3].

Many urban planning and design decisions aimed at improving urban living conditions have been developed in recent years [4]. For example, in the field of air quality, since the year 2000, low-emission zones have been defined in a number of European cities in response to concerns about air pollution and public health. Although such transformations have high political and financial costs, there have been few real-life assessments to date of the well-being and health effects of such environmental policies as well as the role of social health inequalities [5]. Associations between urban changes, environmental exposures, and health-related behaviors generally form a highly complex network involving numerous interactions [6]. More specifically, the mechanisms underlying the associations between changes brought about by urban interventions and changes in environmental risks and health-related behaviors remain understudied. Increased understanding of such mechanisms is needed to clarify the significance and role of specific modifiable determinants.

The natural experiment approach is a way to assess the health impacts of urban interventions and represents an opportunity for innovative research. The Medical Research Council (MRC, United Kingdom) defines a natural experiment as an approach “*which exploit(s) natural or unplanned variation in exposure, i.e., variation that is not manipulated for the purposes of research*” [7, 8]. In a recent “call to action” for the transition to health and sustainable cities, the authors recommended conducting natural experiments to assess the impact of urban interventions on health, social, environmental, and equity outcomes [2]. The natural experiment approach has already been well developed in other research disciplines such as economics. Joshua Angrist, Guido Imbens, and David Card shared the 2021 Nobel prize in economic sciences for their research based on this approach. They showed how causality can be inferred from observational data in real-world natural experiments, specifically regarding labor market issues. In other words, the natural experiment approach takes advantage of the circumstances in which relevant changes occur in a given area and population, e.g., an urban intervention, and then tries to plausibly attribute changes in outcomes of interest to the intervention [9]. Natural experiments are increasingly considered as being able to provide important new input in research and can be very useful for informing policy decisions [10].

In a recent systematic review, 15 studies describing natural experiments using changes in the built environment and investigating health-related consequences were identified. Eight of these reported (favorable) changes in active mobility and dietary intakes when measured on residents after one year post-baseline [4]. However, the authors highlighted that it is difficult to draw overall conclusions, especially because of methodological limitations such as a lack of a comparison group or limited sample sizes.

The main objective of the UrbASanté study is to assess how urban interventions can modify environmental exposures (including air quality, noise, food environment, built, and social characteristics), health-related behaviors, and self-reported health using a natural experiment protocol in Paris (France).

The specific aims of the project are (i) to develop a combination of methods to assess and monitor environmental exposures, health-risk behaviors, and self-reported health; (ii) to collect relevant data during experimentation at a local level in a real-life setting; and (iii) to analyze the data generated to better understand the effects of urban transformations on environmental exposures, health-risk behaviors, and self-reported health in order to more accurately guide public health and urban planning policies.

## Methods

### Study design

The study is based on a natural experiment approach with a before/after protocol design to assess changes in environmental exposures, health-risk behaviors, and self-reported health outcomes of a resident adult population before (T0) and after (T1) the implementation of a time series of urban interventions.

**Fig. 1:**
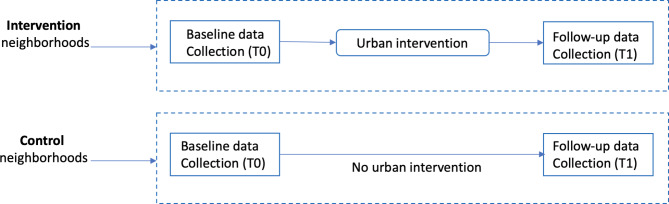
Overall design of the UrbASanté study. (adapted from Leatherdale et al., 2019)

The changes observed in adult residents located in the intervention areas (“exposed population”) will be compared to those in adults living in control areas (“unexposed population”). Control areas are located close to areas with urban intervention sites but are not themselves subject to urban intervention. As described in Fig. 1, the data collection will be implemented in two stages in both the intervention and the control areas: a first stage at baseline (T0, 2022), and a second stage (T1, 2025) after the urban interventions.

#### Intervention and control neighborhoods

**Fig. 2:**
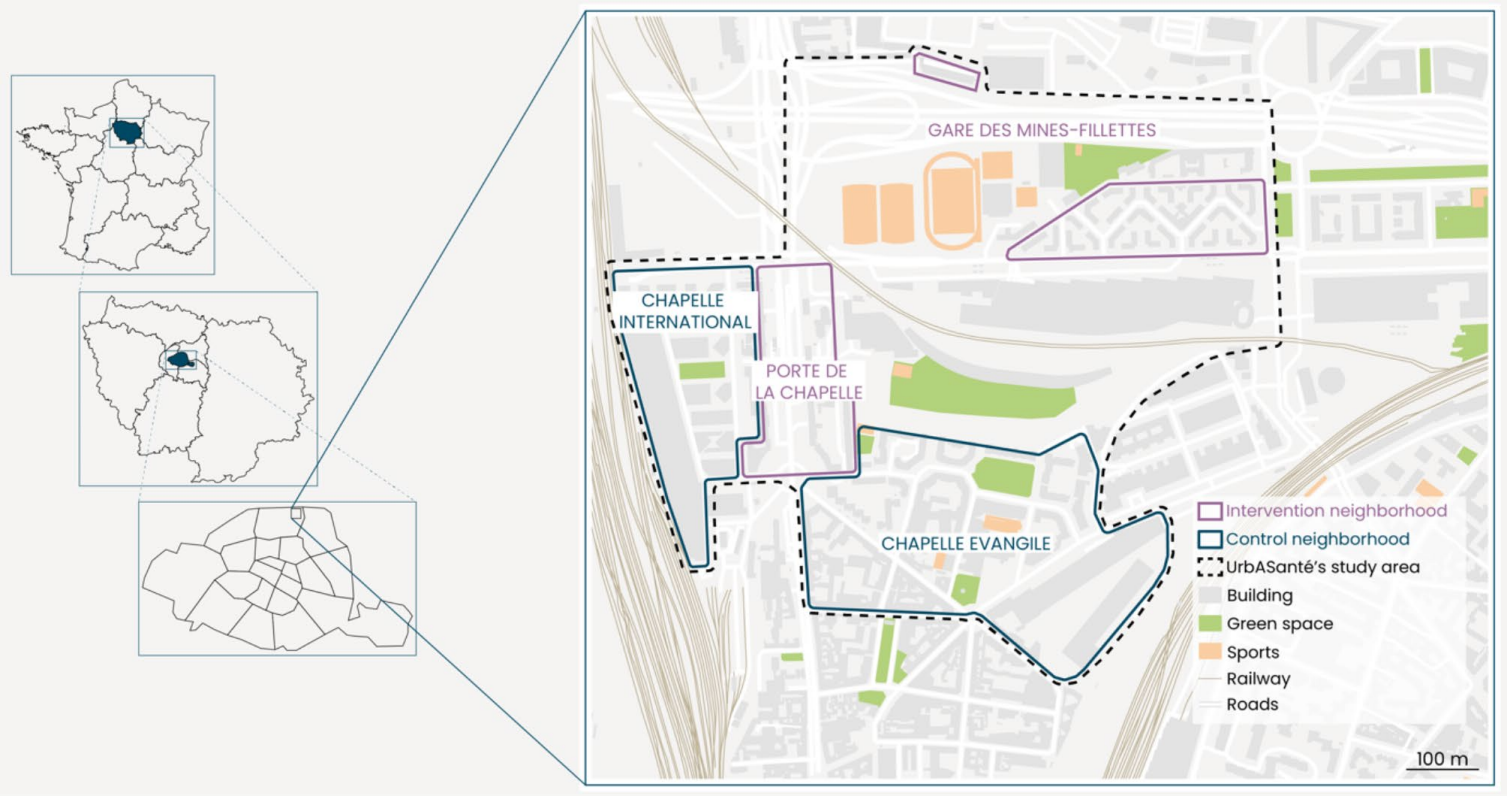
Intervention and control neighborhoods of the study area of the UrbASanté study

The UrbASanté study will examine potential changes in environmental exposures, health-related behaviors, and self-reported health due to urban interventions in an urban area called Porte de la Chapelle in the northern part of the city of Paris (France). This area includes four contiguous neighborhoods located in the northern part of the 18th arrondissement of Paris (Fig. 2).

This area exhibits specific characteristics compared to the broader Parisian conurbation: a large part of its population is socioeconomically deprived and it is exposed to high levels of pollutants [11]. This area is also defined as a place of interest in the context of the Olympic and Paralympic games scheduled to take place in Paris in 2024. Urban interventions will occur with different stages of transformation from 2024 (the Paris Olympic and Paralympic games) to 2030 (legacy of the Paris Olympic and Paralympic games) depending on each neighborhood.

Two neighborhoods will be referred to as “intervention” neighborhoods, with measures taken before and after a series of urban changes that will start in 2023 (Fig. 2).

- The main urban changes in the Gare des Mines-Fillettes neighborhood (which includes the two urban sections of Charles Hermite and Valentin Abeille) will comprise the creation or renewal of urban green areas, pedestrian/cycle pathways and urban corridors, housing, redesigning of public spaces, and more specifically the construction of an Olympic sports facility.
- The urban changes in Porte de la Chapelle Avenue will mainly comprise the development of a new public transport network (bus) as well as the inclusion of cycle and pedestrian pathways and the widening of sidewalks.

Two neighborhoods will be referred to as “control” neighborhoods, with measurements at the same time periods as for the “intervention” neighborhoods.

- Chapelle-international is a new neighborhood built on the site of a former railway wasteland. At the end of 2019, more than 1,500 residents had already moved into this neighborhood.
- The Chapelle-Evangile neighborhood will not undergo specific urban interventions, except in the northern part, where the renovation and expansion of a green area are currently being finalized.

#### Resident data collection

In all studied neighborhoods, the criteria for inclusion will comprise: being an adult (18 years of age or older); residing in the neighborhood; and being able to read, write, or understand French well enough to complete the UrbASanté questionnaire (online or on paper) independently or with assistance from a research assistant. The UrbASanté study is conducted in accordance with the guidelines laid down in the Declaration of Helsinki, and all procedures have been approved by the Institutional Review Board of the French Institute for Health and Medical Research (CEI/IRB INSERM no. IRB00003888, IORG0003254, FWA00005831) and registered with the Commission Nationale de l’Informatique et des Libertés (2220971v0). All participants will provide informed consent. Participants will receive a 15 € voucher for returning a fully completed questionnaire.

#### Recruitment plan

We will use posters, flyers, social media, and local newspaper advertisements to raise awareness of the survey and increase enrollment, and we will develop a door-to-door recruitment protocol [12]. In a first step, the research team will identify the social housing providers, building superintendents for social housing buildings, and housing organizations for private buildings to be contacted prior to the field data collection. In a second step, we will set up appointments with building superintendents or private housing organizations to place posters announcing the study inside the buildings, and then a few days later, we will conduct the door-to-door surveys. The door-to-door surveys will aim to explain the UrbASanté study, distribute flyers and questionnaires, and collect email addresses for follow-up with inhabitants expressing interest in the research. In addition, we will organize information and survey sessions in the main lobby of the main buildings.

We will participate in a variety of community events and use social facilities (e.g., the local library) to conduct drop-in sessions, distribute the study questionnaire, and collect email addresses.

#### Mixed-method framework

**Fig. 3:**
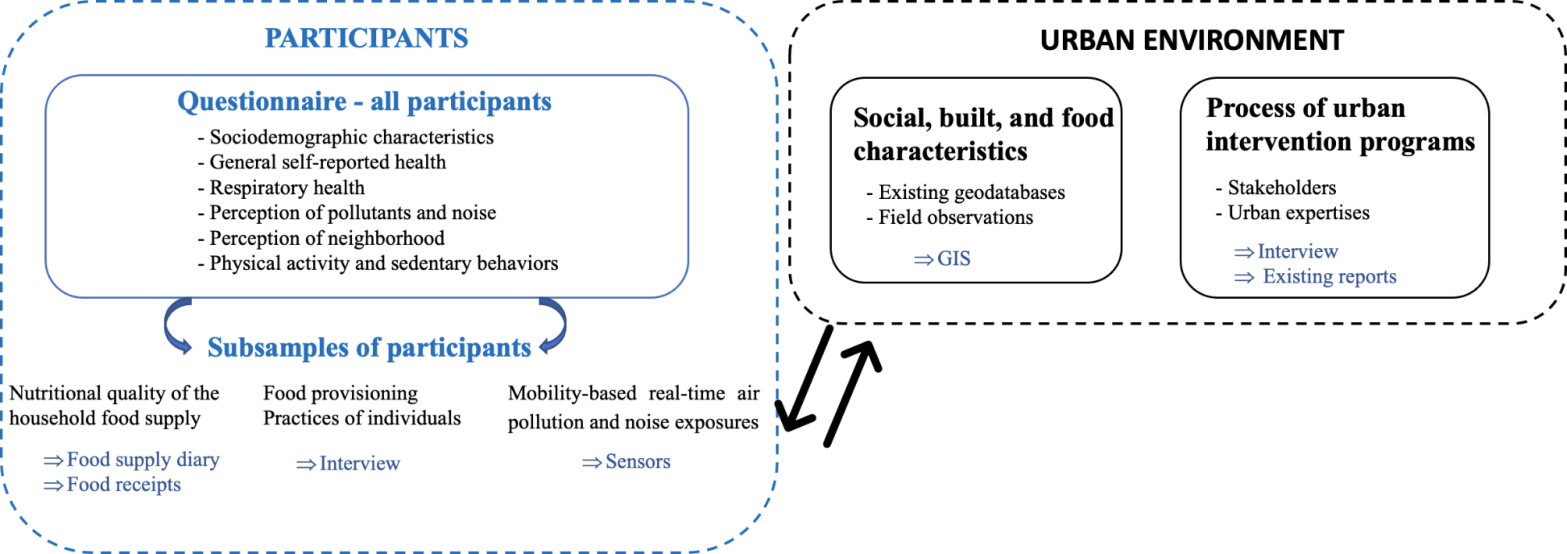
Flowchart of the survey in the UrbASanté study

Our analyses will make use of (i) collection of data relating to exposures and health-related outcomes among all participants and in subsamples, (ii) interviews with residents about their perceptions of their neighborhood and interviews with key stakeholders about the urban change processing, and (iii) existing geodatabases and field observations to characterize the built, social, and food environments (Fig. 3).

## Data collection

### Sampling plan

According to the 2018 census data from the INSEE (the French National Institute for Statistics and Economic Studies), 13,025 adults (≥ 18 years of age) reside in the entire study area (including the control and intervention neighborhoods). We will aim to recruit 600 adult participants at baseline to form the core data survey groups (300 in the intervention area and 300 in the control area). Although there is little precedent for sample size calculations for natural experiment studies (Kestens et al., 2019), potentially due to the multidimensionality of urban interventions, this sample size would be sufficient to assess a change in health-risk behaviors in the context of urban redevelopment. Indeed, assuming a power of 90% and an alpha of 0.05, 380 participants will be required to detect the effect reported by Pazin et al. (2016) for instance, i.e. an increase of 32 (95% CI: 15–51) min/week of walking after development of a new walking and cycling route [13].

In addition, we will aim to define three subsamples of participants to assess:

- the nutritional quality of the household food supply through a food supply diary for a subsample among 400 participants (200 in the intervention area and 200 in the control area);
- the food provisioning practices of individuals through a semi-structured interview for a subsample of 30 participants (15 in the intervention area and 15 in the control area);
- the mobility-based real-time air pollution and noise exposures through sensor measurements for a subsample of 30 participants (15 in the intervention area and 15 in the control area).

### Questionnaire (core data survey)—all participants

A questionnaire-based survey (paper and online versions) will be used to collect self-reported information from individuals regarding socio-demographic characteristics, health outcomes (general health, respiratory health, and weight status), physical activity and sedentary behaviors, perception of neighborhood characteristics, air quality, and noise exposure. The questionnaire includes standardized questions derived from existing validated questionnaires. The questionnaires, the coding procedures, as well as the results of the analyses, will be posted online following an open science strategy using the resources of the Very Large Research Infrastructure called Huma-Num (https://www.huma-num.fr/about-us/), which is supported by the French National Center for Scientific Research (CNRS).

#### Health outcomes

Self-reported general health will be assessed by the question “How is your health in general? Is it…” (very good/good/fair/bad/very bad). This question is recommended by the WHO as a standard and cost-effective measure in health surveys [14] and is used in national [15] and European longitudinal studies (e.g., the European Health Interview Survey—EHIS). In addition, standardized questionnaires that are widely used in respiratory epidemiological studies in adults will be used (the European Community Respiratory Health Survey—ECRHS [www.ecrhs.org/] and the Epidemiological Study of the Genetics and Environment of Asthma—EGEA [egeanet.vjf.inserm.fr]). To assess weight status, the participants will be asked to self-report their weight and height in order to calculate their body mass index (BMI = weight [kg] divided by height [meters] squared).

#### Physical activity, active mobility, and sedentary behaviors

Context-specific active mobility, general physical activity, and sedentary behaviors will be evaluated by questions from the Sedentary, Transportation, and Activity Questionnaire (STAQ) [16, 17]. Perception of active modes (walking and cycling) and characteristics of the neighborhood related to active mobility will also be part of the questions asked to the participants.

#### Perception and use of neighborhood

The perception and use of urban public spaces (such as streets and sidewalks, parks and public-green spaces, and recreational areas) will be assessed by questions from a Daily Life Environment questionnaire (Questionnaire sur l’environnement de vie quotidien, QEVIC) [18]. Specific questions will be also added to include the perception of the food environment (presence of food outlets/restaurants) and details of where, when, and why food outlets were chosen.

### Subsamples of participants

#### Food supply subsample

Participants in the food supply sample will be provided with a short food supply diary to record the details of their household food supplies over a month and asked to save the corresponding grocery store and supermarket receipts, which will be used to accurately assess household food expenditures for food groups. Based on the share of expenditures by food group, the nutritional quality of the household food supplies will be assessed using the revised Healthy Purchase Index (r-HPI) described elsewhere [19]. Briefly, the r-HPI is an index obtained by summing a purchase diversity subscore and a purchase quality subscore, ranging from a minimum score of minus 8 to a maximum score of 17 points, where a higher score reflects a higher quality of the household’s monthly food purchases.

#### Subsample of the food provisioning practices of individuals

Food provisioning practice will be assessed with semi-structured interviews to improve the understanding of the changes over the period of urban intervention regarding the food provisioning practices of individuals (providing details regarding the choice of food outlets and how they justify their shopping practices). All interviews will be fully recorded and transcribed by a professional transcription company or by a member of the research team.

#### Mobility-based real-time air pollution and noise exposure subsample

Pollutant concentrations and noise levels will be measured for one week along the daily itineraries of participants with mobile sensors and time-activity diaries. Particulate matter concentrations (PM_2.5_ and PM_10_ fractions), the temperature, and the relative humidity will be measured with AirBeam3 sensors (HabitatMap, New York, USA), while the nitrogen dioxide (NO_2_) and ozone (O_3_) concentrations will be determined with Cairsens® (ENVEA group, Poissy, France) data loggers that integrate reliable electrochemical gas sensors. Noise levels will be measured using noise dosimeters developed by BruitParif (France). The following noise indicators will be calculated: LAeq, LCeq, LAFmax, and LCFmax data.

### Environmental characteristics and urban interventions

#### Characteristics of built, social, and retail food environments with GIS

Existing geodatabases provided by French public institutions will be used to characterize the social (e.g., population census-INSEE), built (e.g., land-use-IGN), and retail food environments of the study area. For example, the characteristics of the built environment of interest will include the presence of green and blue spaces, street networks, amenities, and pedestrian and cycling infrastructures. Social deprivation can be characterized at the local level based on census variables (housing composition and unemployment rate). The food and built environment characteristics will be completed by field observations (e.g., pedestrian and cycling networks, outlet locations). In addition, we will examine urban and social changes in the study areas and more specifically the possibility of a gentrification effect (e.g., displacement of traditional low-income residents by more affluent households) using census data and field observations.

#### Decision-making process of urban interventions

Semi-structured interviews will be conducted among key stakeholders to examine the follow-up of the decision-making process related to urban interventions. The process of designing the urban intervention program (e.g., which parties are involved, how, and when) will be analyzed, as well as the content of the urban planning strategy, in particular its relevance with regard to health-related outcomes. For the relevant planners involved in the project, the interviews will focus on the genesis and progression of the urban projects as a part of the assessment of the decision-making process. Existing reports such as urban diagnoses and urban expertise (grey literature) related to the study areas and provided by local practitioners and urban planners will be gathered to obtain a better understanding of the spatial context in terms of populations, economy, and urban history.

### Planned statistical analyses

The distributions and correlations among environmental exposures, health-related behaviors, and self-reported health outcomes will be studied at each time point (T0 and T1). The between-period differences will be quantified. Furthermore, we will analyze both cross-sectional (at T0 and T1) and longitudinal dimensions (from T0 to T1) using cluster analyses (e.g., *K-*means analysis) and model-based methods (e.g., mixture modeling techniques or latent class growth analysis) [20].

The associations between environmental exposures and health-related behaviors will be examined using each indicator individually or using the profiles by multivariate regression and spatially explicit models. For instance, multilevel regression models will be performed to estimate associations between changes in the environment (air quality, noise, built, social, and food) and changes in health-related behaviors (food supply and habitual active mobility). Other approaches will be considered to strengthen the impact attribution of the urban intervention to the observed changes. For instance, interrupted time series regressions will be considered for environmental exposures assessed continuously at evenly spaced intervals over time with one well-defined change point (i.e., urban interventions).

The difference-in-difference approach will be used to compare the changes in the outcomes among the participants exposed to the intervention with the change among those who remain unexposed (i.e., the control group).

Additionally, time sequences of the observations (“life-segment”) will be generated for participants included in subsamples. The dynamic processes interrelating the exposures (air quality, noise, social, built, and food), the places visited, and the health-risk outcomes will be examined by using space-time disaggregation of the data: case-crossover analyses allowing each person to be compared with themselves will thus be conducted. Possible effect measure modification by socioeconomic status will be explored using interaction terms and stratified models. Particularly, the reasons why people do or do not use local food outlets and how low-income participants access and perceive the food environment compared to more advantaged segments of the population will be examined.

## Discussion

### An interdisciplinary and collaborative work at various levels

The UrbASanté study represents an innovative scientific interdisciplinary effort and aims to estimate longitudinal relationships in the interaction between urban characteristics and health-risk behaviors and self-reported health, and more specifically to investigate the processes that may lead to a healthier and more sustainable city. The project will, therefore, be carried out through the collaborative work of a consortium ensuring complementarity between geographers, epidemiologists, experts in atmospheric physics, urban planners, and stakeholders in the field of urban planning and public health.

The objectives of UrbASanté will be achieved through close interaction, with a participatory research dynamic, between local stakeholders involved in urban planning and public health and the research community in a type of “action research”. Paris city stakeholders have been involved in the research consortium from the start of the project to provide relevant information regarding research (questions and methods) and local experiences and to facilitate evidence-based decision-making. Overall, the UrbASanté study will contribute to “breaking the silo approach” that tends to exist in urban planning issues involving different urban services, approaches, and disciplines.

In addition, the first stage of our fieldwork will be to both identify and meet local stakeholders and practitioners, community and association leaders, and building superintendents to explain the how and why of the UrbASanté research project and that the results that it is expected to yield may be of importance to the residents. We will then participate in the life of the neighborhoods (e.g., by becoming involved in local events and activities) to become known to the residents and to facilitate (future) door-to-door surveys.

This process of communication with all local stakeholders also allows for a better understanding of local situations, and additional city and local knowledge users will be included as the research unfolds. This back-and-forth communication between city/local parties and researchers will allow the project to be improved and it may also facilitate dissemination of the results at a later stage.

### Study design

A particular methodological challenge for our study is the recruitment of participants in a context of (i) low socioeconomic neighborhoods with deprived populations who are generally a hard-to-reach segment of the overall population for research purposes and (ii) a hot topic (urban changes and health) in “hot places” that receive a great deal of political and urban planning attention in the context of ongoing large urban regeneration programs, especially with the perspective of the Olympic and Paralympic Games taking place in Paris in 2024.

An additional methodological challenge is the ability to identify intervention and control neighborhoods. Ideally, this involves finding similar intervention and control neighborhoods, with the only difference being that one or several of the neighborhoods will undergo an urban change. In practice, none of the neighborhoods are equally matched in terms of all of the characteristics of interest. In our study, the main objective criterion to identify equally matched neighborhoods is the socioeconomic level, which will be used to define disadvantaged vs. more advantaged areas. This design is appropriate for our study because the intervention and control sites are in the same residential area, with urban intervention taking place in specific neighborhoods. While major transport infrastructures or those related to the Olympic Games will benefit all neighborhoods and more broadly all of Paris and its surrounding areas, specific urban interventions (i.e., housing, street, site) are restricted to specific neighborhoods only.

### Combination of methods

We pay particular attention to collect subjective and objective measurements of environmental characteristics of interest [21]. When attempting to explain health-related behaviors in terms of impacts of the environment, it is important to capture both objective and subjective assessments, in other words how the environment is perceived by those who inhabit it [22, 23]. The perceptions of residents are typically obtained through interviews or self-administered questionnaires, as well as objective measures derived from field observations, sensors, and existing geospatial data.

### Limitations and scientific risks

The UrbASanté research project involves several risks. The first risk lies with the defining feature of a natural experiment, namely that the implementation of the urban intervention is not under the control of the research consortium. We are aware that the project depends on the schedule of the planned urban changes and, therefore, remains highly dependent on variables external to the project. However, this risk is reduced by involvement of the stakeholders as full partners in our consortium, thereby ensuring visibility of the steps taken to implement urban changes in the field. In addition, the urban interventions used as support of the natural experiment are included in major urban planning projects in the city of Paris.

Most of the health-related behaviors and health outcomes are self-reported. This represents a limitation for measurements associated with smoking, physical activity, and sedentary behaviors because of known social desirability bias [24, 25]. However, self-perceived health has received increasing interest in international studies because it correlates strongly with objective measures of health and it is consistent with predictions of future health problems and mortality [26]. Self-perceived health is also a widely used measurement to study trends and inequalities between genders, as well as across population groups [27].

Assessment of the effects of urban changes at the neighborhood level provides a unique opportunity to generate insights regarding the range and the nature of both positive and negative impacts on health-related outcomes. It should help elucidate some of the specific components and mechanisms through which urban changes can influence health-risk behaviors.

The results will be of major relevance for research and local public policies (i) in the design of healthy cities and (ii) to maximize the benefits of urban interventions for which quantitative scientific knowledge is largely insufficient compared to the substantial societal expectations.

Urban planning and public health authorities need tools to recognize opportunities, support decision-making, provide data to support funding applications, and demonstrate the impact of their initiatives to embed health into urban policy. The results obtained will contribute to filling gaps in major research areas, since the project is fully in line with the objectives of the “Health in All Policies”, developed as a key initiative of the WHO Healthy Cities Network to provide practical strategies for integrating health considerations into all government policies, not only within the health portfolio.

## Data Availability

Not applicable—the collection of data is ongoing.

## Abbreviations

BMI: Body Mass Index
EHIS: European Health Interview Survey
ECRHS: European Community Respiratory Health Survey
EGEA: Epidemiological Study of the Genetics and Environment of Asthma
FHS: Framingham Heart Study
INSEE: National Institute of Statistics and Economic Studies (France)
IGN: National Institute of Geographic and Forest Information (France)
Leq: equivalent continuous sound level is the sound level in decibels, LAeq is the A-weighted leq sound level (A-weighting is the ‘common’ name for *frequency-weighted* sound levels, measured over the ‘A’ frequency range), LCeq is the C-weighted leq sound level (C-weighted is used for high-level measurements).
LAFmax: A-weighted, maximum sound level measured with a fast time constant
LCFmax: C-weighted, fast response, maximum sound level
PM: Particulate matter, (PM_10_, particles with diameters that are generally 10 micrometers or smaller; PM_2.5_, fine particles with diameters that are generally 2.5 micrometers or smaller; and PM_1_, ultrafine particles with diameters that are generally 1 micrometer or smaller)
STAQ: Sedentary, Transportation, and Activity Questionnaire
WHO: World Health Organization
